# The Relationship Between Different Aspects of Theory of Mind and Symptom Clusters in Psychotic Disorders: Deconstructing Theory of Mind Into Cognitive, Affective, and Hyper Theory of Mind

**DOI:** 10.3389/fpsyt.2021.607154

**Published:** 2021-04-09

**Authors:** Laura M.-L. Dorn, Nele Struck, Florian Bitsch, Irina Falkenberg, Tilo Kircher, Winfried Rief, Stephanie Mehl

**Affiliations:** ^1^Department of Clinical Psychology and Psychotherapy, Philipps-University Marburg, Marburg, Germany; ^2^Department of Psychiatry and Psychotherapy, Philipps-University Marburg, Marburg, Germany; ^3^Center for Mind, Brain and Behavior, Philipps-University Marburg, Marburg, Germany; ^4^Department of Health and Social Work, Frankfurt University of Applied Sciences, Frankfurt, Germany

**Keywords:** psychosis, delusion, cognitive biases, theory of mind, social cognition, Frith-Happé animations

## Abstract

**Background:** Several meta-analyses highlight pronounced problems in general Theory of Mind (ToM), the ability to infer other persons' mental states, in patients with psychosis in comparison to non-clinical controls. In addition, first studies suggest associations between Hyper-ToM, an exaggerated inference of mental states to others, and delusions. Research on different ToM subtypes (Cognitive ToM, Affective ToM, and Hyper-ToM) and symptom clusters of psychosis (positive, negative, and disorganized symptoms) have gathered conflicting findings. Thus, the present study examined group differences between patients with psychosis and non-clinical controls concerning Cognitive ToM/Affective ToM and Hyper-ToM. Further, the association between ToM subtypes and symptom clusters (positive, negative, and disorganized symptoms) were examined.

**Methods:** Patients with psychotic disorders (*n* = 64, 1/3 with present delusions indicated by a minimum score of four in the *PANSS* P1 item) and non-clinical controls (*n* = 21) were examined with assessments of Cognitive ToM and Affective ToM abilities and Hyper-ToM errors using the Frith-Happé animations. Psychopathology was assessed using the Positive and Negative Syndrome Scale.

**Results:** Patients with psychosis presented more pronounced problems in Cognitive and Affective ToM in comparison to non-clinical controls, whereas there were no group differences with regard to Hyper-ToM errors. Furthermore, deficits in Cognitive ToM were associated with general delusions, whereas problems in Affective ToM were associated with negative and disorganized symptoms. In addition, there was no association between Hyper-ToM errors and any symptoms when controlling for years of education.

**Conclusions:** Our findings suggest that deficits in ToM subtypes might not be directly related to delusions and positive symptoms and are in line with more recently developed cognitive models of delusions. In addition, our results support the well-established finding of associations between ToM alterations and negative or disorganized symptoms. Our results shed light on the role of different dimensions of ToM in specific symptoms of psychosis.

## Introduction

Theory of Mind (ToM) is defined as the ability to infer other persons' mental states, including their thoughts, intentions, and emotions (1). Frith (2) was the first one to link problems in ToM with psychosis, and he postulated ToM deficits as a predisposing cognitive factor for delusions. Delusions are defined as abnormal beliefs that are believed with absolute conviction, experienced as self-evident truths, and not modifiable by experience (3). Deficits in ToM are one out of five core components of social cognitions (ToM, social perception, social knowledge, attributional bias, emotional processing) (4) and have since then been widely studied in patients with psychotic disorders (5). Social cognition is defined as ‘the mental operations that underlie social interactions, including perceiving, interpreting, and generating responses to the intentions, dispositions, and behaviors of others’ (4). Thus, deficits in ToM are closely related to problems in social functioning (6–8) and a lower quality of life in patients with psychosis (8).

Several meta-analyses indicated deficits in the overall ToM ability in psychotic patients, their ToM performance was on average more than one standard deviation below the performance of non-clinical controls (9–12). In addition, problems in ToM depend on patients' phase of illness: patients with an acute psychotic episode show more pronounced ToM deficits in comparison to patients with remitted symptoms of schizophrenia (9). Thus, ToM deficits were intensely discussed within theoretical models of psychosis as a potential risk factor (13–17).

To investigate how ToM deficits in individuals are associated with specific symptoms of psychosis, several earlier studies subdivided the symptoms of patients with psychosis into symptom clusters: symptoms of disorganization, positive symptoms (“reality distortion”), and negative symptoms (18). In a recent meta-analysis that summarized these findings, patients with disorganized symptoms were most impaired in ToM, followed by patients with negative symptoms and then patients with positive symptoms (11, 18). Thus, ToM in general as a cognitive correlate of symptom clusters in psychosis is well studied, but we know little about specific associations between ToM and psychotic symptom subdomains as negative or positive symptoms (e.g., delusions and hallucinations).

Concerning the relationship between specific positive symptoms such as delusions (of persecution) and ToM, several studies summarized in a review (17) found correlations between ToM deficits and more pronounced persecutory delusions (19–22) and general delusions (21), whereas other studies did not find an association (23, 24). In their review, Garety and Freeman assumed that about half of the studies found associations between problems in ToM and delusions, whereas the other half of the studies did not report associations and this observation led the authors to exclude ToM from their current theoretical models of the formation and maintenance of delusions (16).

One explanation for these inconsistent findings might be the fact that a large number of previous studies that assessed ToM deficits in patients with psychosis used a simple dichotomous right-or-wrong answer format. In these studies, wrong answers were typically interpreted as reduced ToM/undermentalizing. In terms of reduced ToM, it seems necessary to distinguish between deficits in more cognitive or more affective ToM abilities, which could lead to a differentiated understanding of the association between psychotic symptoms and reduced ToM subtypes. Whereas, cognitive ToM requires a cognitive understanding of the other person's mental state including their thoughts and intentions, affective ToM is defined as an empathic evaluation of the other person's emotional state (25). Nevertheless, only a small number of studies investigated cognitive and affective ToM separately in patients with psychosis (21, 25, 26). Results of these studies indicate that deficits in cognitive ToM were associated with positive symptoms (21, 26), whereas problems in affective ToM were linked with more pronounced negative symptoms (25, 26). Thus, these findings suggest that both ToM abilities may be involved in different cognitive processes, so overall, to better understand the relationship between reduced ToM abilities and specific psychotic symptoms, it is important to consider and assess both reduced ToM abilities: cognitive ToM and affective ToM (27).

An additional explanation for the heterogeneous results regarding the relationship between ToM and positive symptoms/delusions in psychotic patients is the Hyper-ToM/overmentalizing approach according to Frith (2) and Abu-Akel (28), which complements previous research on reduced ToM/undermentalizing. Hyper-ToM is defined as an excessive attribution to other people's state of mind, and this excessive attribution leads to inaccurate conclusions about their mental state (28, 29). Thus, ToM problems can be viewed on a continuum from reduced ToM to Hyper-ToM and both can lead to errors in ToM tasks, but a differential error analysis would reveal these distinct error types. Therefore, the concept of Hyper-ToM has not always been sufficiently considered in previous research, while more recent studies focus increasingly on Hyper-ToM (30–33). Interestingly, Frith proposed that patients with delusions present more problems in Hyper-ToM/overmentalizing in comparison to undermentalization (34). In support of this assumption, the first results suggest an association between Hyper-ToM errors and more pronounced positive symptoms (19, 26, 32, 33) and delusions in particular (19, 26). Thus, Hyper-ToM errors, rather than general deficits in ToM, may play an important role in the formation and maintenance of delusions and positive symptoms of psychosis.

In summary, the present study aims to investigate the relationship between problems in different subtypes of ToM (cognitive ToM, affective ToM, and Hyper-ToM) and various symptom clusters of psychosis (positive, negative, and disorganized symptoms) in a large sample of patients with psychosis using a reliable and valid ToM assessment. In specific, we hypothesized that patients with psychosis are more severely impaired in cognitive ToM and affective ToM and show more pronounced Hyper-ToM errors compared to non-clinical controls (1). We further assumed that those deficits in cognitive ToM are associated with more pronounced positive symptoms and, in particular, with delusions (2), whereas deficits in affective ToM are associated with negative symptoms and symptoms of disorganization (3). In addition, we assumed that more pronounced Hyper-ToM errors are associated with positive symptoms in general and delusions in particular (4).

## Materials and Methods

### Participants

Participants were 64 patients diagnosed with a psychotic disorder (schizophrenia *n* = 54; schizoaffective disorder *n* = 8; delusional disorder *n* = 1; acute psychotic disorder *n* = 1) and 21 non-clinical controls. Inclusion criteria were a psychotic disorder verified by the *Structured Clinical Interview for DSM IV (SCID-IV)* (35). Additional inclusion criteria were age between 18 and 65 years and adequate language skills. Exclusion criteria for patients were the presence of a borderline personality disorder, dementia, or substance use disorder in the last six months (verified by the *SCID-IV* and patient files). Exclusion criteria for the non-clinical controls were a psychotic disorder in their lifetime or another mental disorder within the last ten years (verified by the *SCID-IV*).

### Recruitment and Procedure

Eligible patients were contacted via their attending physicians/therapists and then informed about the study by the study assistant and signed the informed consent form. Non-clinical controls were recruited via notices on public places and mailing lists for University students and matched with the first 21 patients already recruited in terms of age, gender, and educational level (3:1 matching due to inadequate funding). As compensation, patients received a financial payment (20€). Non-clinical controls received either a financial payment (20€) or, if desired, a certificate of attendance to meet their curriculum requirements (e.g., ECTS), as 11 of the 21 non-clinical controls were students. All participants gave a written declaration of informed consent. The present study was approved by the local ethics committee.

In the first session, trained raters conducted the *SCID-IV* interview (35) and the *PANSS* interview [*Positive and Negative Syndrome Scale*, (36)]. In the second appointment, participants filled out questionnaires on sociodemographic data and verbal IQ. Then, a study assistant conducted the *Frith-Happé animations paradigm* (37). Due to the length of the interviews, the patients' assessments took place at two different appointments to avoid concentration problems.

### Instruments

Verbal intelligence was estimated using the German IQ test *Mehrfachwahl-Wortschatztest* [*MWTB*; (38)]. The MWTB is a vocabulary IQ test and consists of 37 tasks, in which the participant is asked to distinguish one target word from four distracting non-words. The authors described the MWTB as a reliable and valid instrument.

Positive symptoms, negative symptoms, and general psychopathology were assessed using the *Positive and Negative Syndrome Scale* (36), a semi-structured interview, in which 30 symptoms are measured on a seven-point Likert scale. The PANSS rating was based on the German version of the Structured Clinical Interview for PANSS (39). The PANSS ratings were carried out by trained raters who received ten training units to conduct and evaluate the PANSS interview. The inter-rater reliability (ICC, corr. *R*^2^) was satisfactory to high (between 0.74 and 0.91). In the statistical analyses, we used the 20-item, five-factor PANSS model proposed by Wallwork and colleagues (40), which presented the best model fit in factor analyses (41) and consists of the factors: positive, negative, disorganized, excited, and depressed factor. In the present study, we used the positive, negative and disorganized symptom factors. In addition, general delusions were assessed with the PANSS item P1 and persecutory delusions were assessed with the PANSS item P6. To ensure that the results we obtained regarding associations between psychopathology and ToM scores were independent of the PANSS factor model, we also used the PANSS factor model proposed by van der Gaag and colleagues (42) and the PANSS negative symptom factors by Liemburg and colleagues (43), the results are provided in the Supplementary Material.

Theory of Mind was assessed using the advanced multiple-choice version (37) of the *Frith-Happé animations paradigm* (44), an objective and standardized test (37). Abell and colleagues (44) developed the test in its original version, which White (37) found to be time-consuming and subjective. Therefore, she developed a more objective and feasible evaluation method through a series of multiple-choice questions, which was used in the present study. The authors identified the Frith-Happé animations as a sensitive and reliable instrument (37).

The Frith-Happé animations consist of twelve short animated videos of two triangles performing three different kinds of movements: (1) they either move randomly and do not seem to interact with each other (random condition), (2) they move in a goal-directed manner and one triangle responds and interacts with the physical actions or behavior of the second triangle (goal-directed condition) or (3) their movements indicate that one triangle infers the mental state of the second triangle and reacts on it (ToM condition) (37). After two practice animation trials including feedback by the experimenter, the videos were presented in a pseudo-randomized order. The participants watched the videos and were then asked to first categorize them in a multiple-choice format either as indicating random movement (RD), goal-directed movement (GD), or movements of the triangles indicating that one triangle reacted on the other triangle's putative mental state (*Cognitive ToM*). If an animation was correctly identified as indicating Cognitive ToM, participants were then asked at the end of the animation to rate the feelings of the triangles towardz each other by selecting the appropriate feeling out of five suggested feelings in a multiple-choice format (*Affective ToM*). To assess Hyper-ToM errors, we developed an additional scoring, comparable to previous studies (45). A weighted score with a maximum total score of 12 was determined, consisting of eight possible errors in categorizing random videos as either goal-directed (score = 1) or as ToM (score = 2) or goal-directed videos were categorized as ToM (score = 1), these incorrect categorizations were classified as *Hyper-ToM errors*.

### Statistical Analyses

Analyses were conducted using SPSS for Windows (Version 25). Outlier analysis was performed using boxplots. The data were then winsorized, with outliers being replaced by the next highest value in the sample that was not identified as an outlier (46).

Concerning all statistical analyses, the assumed significance level was set at *p* < 0.05. Analysis of the data distribution showed that ToM data and data of *PANSS* symptom factors were not normally distributed, as determined by the Shapiro-Wilk test. In addition, since the range of test scores of the Frith-Happé animations was rather small, ToM data were analyzed using non-parametric tests, as recommended by the test authors (37). Before group comparisons, we checked whether variances are homogenous using Levene tests. If results of Levene tests suggested homogenous variances, groups were compared using ANOVAs, even if variables were not normally distributed, as parametric tests present more pronounced statistical power compared to non-parametric tests (47). If Levene tests suggested heterogeneous variances, we used non-parametric tests.

First, patients with psychosis and non-clinical controls (NC) were compared with regard to sociodemographic and clinical variables using either univariate ANOVAs or Mann-Whitney-*U*-tests depending on preconditions, as outlined above. Chi^2^ tests were performed to compare the groups in nominal data. In case of statistically significant group differences, we analyzed whether the specific variables were related to Cognitive ToM/Affective ToM/Hyper-ToM, using Pearson correlation coefficients or Spearman correlation coefficients (two-tailed) (depending on the distribution of the data). If there were statistically significant correlations, these variables were included as covariates in further analyses.

Second, patients with psychosis and non-clinical controls were compared in Cognitive ToM/Affective ToM and Hyper-ToM (hypothesis 1), using ANCOVAs, controlling for group differences in sociodemographic data. In case of statistically significant Levene tests, we performed a non-parametric or rank analysis of covariance [ “Quade's test,” (48)], which included three steps: First, we transformed ToM scores and data of the covariate to rank data, using the default settings in the SPSS RANK procedure. Second, we performed a linear regression analysis using the rank data of the ToM scores as dependent variable and rank data of the covariates as independent data and saved the unstandardized residuals of the dependent variable. Third, we performed an ANOVA, using the residual data as the dependent variable and the variable group (patients and non-clinical controls) as the criterion variable.

Third, we examined bivariate correlations to investigate the relationship between Cognitive ToM/Affective ToM and symptoms (positive, negative, and disorganized symptoms; general/persecutory delusions) using either Spearman correlation coefficients or Pearson coefficients depending on the presence/absence of normally distributed variables (two-tailed, hypothesis 2 and 3). Finally, we investigated the association between Hyper-ToM errors and delusions (general/persecutory delusions) and positive, negative, and disorganized symptoms using Pearson or Spearman correlations (two-tailed, hypothesis 4). In addition, in the case of statistically significant Levene tests, we performed partial rank correlation analyses to control for group differences in sociodemographic data, which included two steps: First, we performed a non-parametric Spearman rho correlation analysis between ToM scores and symptom data and saved the variables of Spearman rho correlations as the current data set. Second, we computed partial correlations using these correlation variables as the input data and sociodemographic variables as covariates.

## Results

### Sample Characteristics

Table 1 depicts sociodemographic and clinical variables of patients with psychosis (*n* = 64) and non-clinical controls (*n* = 21). Twenty two of the patients (34.4%) were recruited in an inpatient unit, 32 patients (50%) in an outpatient treatment center, and eight additional patients (12.5%) were recruited via public advertisement. About half of the patients (55%) were female; the mean age was 37.5 years. The mean years of education in the patient group was 13.7 years, and the highest level of education was a graduate degree. Patients with psychosis reported a relatively long duration of their psychotic illness (mean score: 14 years) and a mean number of six psychotic episodes. 41% of the patients were in a remitted phase of their psychotic disorder as indicated by Andreasen (49) and about one-third of the patients (*n* = 24) had acute delusions indicated by a minimum score of four in the *PANSS* (36) P1 item (general delusions).

**Table 1:** Sociodemographic and clinical characteristics of patients with psychosis and non-clinical controls.

	**Patients with psychosis (*n* = 64)**	***n***	**Non-clinical controls (NC) (*n* = 21)**	***n***	**Test statistics**
**Demographic variables**	***M (SD)***		***M (SD)***		
Age (years)	37.5 (13.2)	64	36.10 (13.15)	21	*F*_(1, 83)_ = 0.17, *p* = 0.68
Gender					
Males *N* (%)	35 (45.3)	64	15 (71.4)	21	χ2_(1)_ = 1.83, *p* = 0.18
Females *N* (%)	29 (54.7)		6 (28.6)		
Years of Education	13.7 (4.5)	62	18.6 (5.4)	20	***F***_**(1, 80)**_ **= 16.37**, ***p*** < 0.001** patients < HC
IQ (MWTB)	105.2 (13.5)	64	111.29 (16.05)	21	*F*_(1, 83)_ = 2.93, *p* = 0.09
Clinical variables					
Duration of illness (years)	14.1 (10.1)	53	–	–	
Psychotic episodes (number)	5.9 (6.9)	57	–	–	
Age of onset of psychotic disorder (years)	24.6 (10.1)	41	–	–	
PANSS positive symptom factor (40)	9.33 (3.77)	63	–	–	
PANSS negative symptom factor (40)	12.38 (5.04)	63	–	–	
PANSS disorganized symptom factor (40)	5.48 (2.09)	63	–	–	
PANSS total score	59.5 (14.6)	63	–	–	
Andreasen's remission rate *N* (%)	26 (40.6)	64	–	–	

*M, Mean; SD, Standard deviation; MWT-B, Mehrfachwahl-Wortschatztest (38); PANSS, Positive and Negative Syndrome Scale (36); Remission rate defined according to Andreasen (scores of the PANSS items P1, P2, P3, N1, N4, N6, G5, and G9 item ≤ 3, respectively) (49); One patient perceived the PANSS interview as too stressful, and interrupted the interview, PANSS scores are partially missing for one person. Statistical significance is indicated by bold values*.

There were no statistically significant group differences between patients with psychosis and non-clinical controls with regard to age [*F*_(1, 83)_ = 0.17, *p* = 0.68], gender [$\chi_{\left(1\right)}^{2}$ = 1.83, *p* = 0.18], and estimated verbal IQ [*F*_(1, 83)_ = 2.93, *p* = 0.09]. Results of an univariate ANOVA indicated statistically significant group differences with regard to years of education [*F*_(1, 80)_ = 16.37, *p* < 0.001]. In the next step, associations between years of education and ToM variables (Cognitive ToM, Affective ToM and Hyper-ToM) were tested, using Spearman correlation coefficients. There was a statistically significant association between Hyper-ToM errors and years of education (*r*_s_ = −0.33, *p* = 0.02), whereas all other associations were not statistically significant [Cognitive ToM (*r*_s_ = 0.23, *p* = 0.07); Affective ToM (*r*_s_ = 0.15, *p* = 0.24)]. Thus, all further analyses on Hyper-ToM were controlled for years of education.

### Group Comparisons in Cognitive ToM, Affective ToM, and Hyper-ToM

The results of the group comparisons between patients with psychosis and non-clinical controls (NC) are depicted in Table 2. Group comparisons were performed as Mann-Whitney-*U* tests, as all ToM variables were not normally distributed and Levene tests suggested heterogeneous variances. Results indicated that patients with psychosis were more severely impaired in both Cognitive ToM (*U* = 444.0, *z* = −2.37 *p* = 0.02) and Affective ToM (*U* = 412.0, *z* = −2.69 *p* = 0.01) in comparison to the non-clinical controls. Group comparisons in Hyper-ToM were performed as “Quades test” and results suggested no statistically significant group differences [*F*_(1, 73)_ = 0.017, *p* = 0.90].

**Table 2:** Comparisons between patients with psychosis and non-clinical controls in Cognitive ToM, Affective ToM, and Hyper-ToM errors.

	**Patients with psychosis (*n* = 64)**	**Non-clinical controls NC (*n* = 21)**	**Test statistics Mann-Whitney-*U***
**Frith-Happé animations**	***M (SD)***	***M (SD)***	
Cognitive ToM	8.88 (1.90)	9.81 (2.02)	***U*** **= 444.0**, ***z*** **= −2.37** ***p*** **= 0.02 Patients < NC**
Affective ToM	3.69 (2.11)	4.86 (1.35)	***U*** **= 412.0**, ***z*** **= −2.69** ***p*** **= 0.01 Patients < NC**
Hyper-ToM	2.46 (1.78)	1.95 (1.79)	*F*_(1, 73)_ = 0.017, *p* = 0.90

*M, Mean; SD, Standard deviation; U, Mann-Whitney-U test; F, “Quades test”; Scoring, For the multiple-choice cognitive ToM, a total score of 12 was the maximum, divided into a maximum of four for each of the animation types. Participants could score a total of 8 for the multiple-choice Affective ToM, corresponding to two possible correct answers for each of the ToM animations. According to previous studies (45), we developed an additional scoring to assess Hyper-ToM. A weighted value was determined with a maximum total score of 12, consisting of eight possible errors when categorizing random videos in goal-directed (value = 1) or in ToM (value = 2) and goal-directed videos in ToM (value = 1). Statistical significance is indicated by bold values*.

### Association Between Cognitive ToM, Affective ToM, Hyper-ToM, and Psychotic Symptoms

The results of Spearman correlation analyses are depicted in Table 3. As hypothesized, there was a statistically significant correlation between poorer Cognitive ToM performance and more pronounced general delusions (PANSS P1; *r*_s_ = −0.299, *p* = 0.02). Cognitive ToM was neither significantly associated with other positive symptoms (Wallworks' PANSS positive factor: *r*_s_ = −0.196, *p* = 0.12), nor with persecutory delusions (PANSS P6: *r*_s_ = −0.173, *p* = 0.18). There was a statistically significant association between lower scores in Affective ToM and more pronounced negative symptoms (Wallworks' PANSS negative factor: *r*_s_ = −0.332, *p* < 0.01) and disorganized symptoms (Wallworks' PANSS disorganized factor: *r*_s_ = −0.286, *p* = 0.02). Finally, as Hyper-ToM errors were associated with years of education, the association between Hyper-ToM errors and symptoms was controlled for years of education, using a partial Spearman correlation analyses (see Supplementary Table 1 in the Supplementary Material). Results revealed no statistically significant correlation between more pronounced Hyper-ToM errors and disorganized symptoms (Wallworks' PANSS disorganized factor: *r*_s_ = 0.215, *p* = 0.12) if years of education was included as a covariate. In contrast to our hypotheses, there were no statistically significant associations between Hyper-ToM errors and neither positive symptoms (Wallworks' PANSS positive factor: *r*_s_ = 0.151, *p* = 0.28), nor delusions (general delusions, PANSS P1: *r*_s_ = 0.187, *p* = 0.18; persecutory delusions, PANSS P6: *r*_s_ = 0.051, *p* = 0.72).

**Table 3:** Spearman correlation coefficients between Cognitive ToM, Affective ToM, Hyper-ToM, and clinical symptoms in patients with psychosis.

			**Frith-Happé Cognitive ToM**	**Frith-Happé Affective ToM**	**Frith-Happé Hyper-ToM**
	**M**	**SD**	***r_***s***_* (*p*)**	***r_***s***_* (*p*)**	***r_***s***_* (*p*)**
PANSS positive symptom factor (40)	9.33	3.77	−0.196 (0.12)	−0.188 (0.14)	0.169 (0.22)
PANSS negative symptom factor (40)	12.38	5.04	−0.044 (0.73)	–**0.332 (<0.01)**	−0.095 (0.49)
PANSS disorganized symptom factor (40)	5.48	2.09	−0.239 (0.06)	–**0.286 (0.02)**	**0.320 (0.02)**
General delusions (PANSS P1)	2.94	1.31	–**0.299 (0.02)**	−0.234 (0.07)	0.214 (0.12)
Persecutory delusions (PANSS P6)	2.83	1.43	−0.173 (0.18)	−0.183 (0.15)	0.051 (0.71)

*M, Mean; SD, Standard deviation; r_s_, Spearman correlation; p, significance; PANSS, Positive and Negative Syndrome Scale (36). Statistical significance is indicated by bold values*.

### Additional Exploratory Analyses

In additional exploratory analyses, we investigated whether our results might differ if the well-known PANSS factors according to van der Gaag and colleagues (42) were included in the analysis instead of the factor developed by Wallwork (40). Furthermore, we examined the relationship between ToM and the additional negative factors “expressive deficits” and “social amotivation” proposed by Liemburg (43). The results of additional Spearman correlation analyses are depicted in Supplementary Table 2. Exploratory analyses revealed that there were no differences in results if analyses were repeated using the PANSS factors proposed by van der Gaag et al. (42) regarding Cognitive ToM, except for the association between Cognitive ToM and the PANSS disorganized factor. Symptoms of disorganization (PANSS disorganized factor) were associated with Cognitive ToM (*r*_s_ = −0.31, *p* = 0.01). Concerning Affective ToM, in contrast to our previous results, there was no statistically significant association between Affective ToM and negative symptoms [ “social amotivation” (*r*_s_ = −0.16, *p* = 0.22)]. Furthermore, concerning Hyper-ToM, associations between Hyper-ToM errors and the five PANSS factors were comparable.

## Discussion

To the best of our knowledge, the present study is the first study that examined ToM in a sample of patients with psychotic disorders using the more advanced multiple-choice version (37) of the Frith-Happé animations test (44). In the present study, patients with psychosis presented more pronounced deficits in Cognitive ToM and Affective ToM but did not show the expected tendency to Hyper-ToM errors, in comparison to non-clinical controls. Furthermore, our results indicated that deficits in Cognitive ToM were associated with general delusions, while deficits in Affective ToM were associated with negative and disorganized symptoms. Also, there was no statistically significant association between Hyper-ToM errors and any symptoms, if the influence of education was controlled.

Our results regarding patients' deficits in cognitive and affective ToM are in line with various meta-analyses showing a reduced general ToM ability in psychotic patients compared to non-clinical controls (9–11, 18). In addition, Lugnegård and colleagues (50) used an earlier version of the Frith-Happé animation test. In this task, participants' free verbal descriptions of the triangles were evaluated by independent raters. Raters evaluated first on how accurately the description reflected the events in the animation (ToM appropriateness) and then rated whether the participant described the complex, intentional mental states (ToM intentionality). Thus, the ToM scores are more focused on the Cognitive ToM component. In this task, patients with psychosis also were more impaired in comparison to non-clinical controls (12, 50). Furthermore, only a small number of studies (in line with our study) divided ToM into Cognitive and Affective subcomponents and examined both (25, 26). Our results are consistent with one of these studies that used the Movie Assessment of Social Cognition [MASC; (26)], a test presenting videos of social situations, that also reported more severe problems in both Cognitive ToM and Affective ToM in patients with psychotic disorders as compared with non-clinical controls of large effect size (26). Solely in the study of Shamay-Tsoory and colleagues, patients with psychosis showed more problems in affective ToM compared to non-clinical controls, while there were no differences between both groups in cognitive ToM in a ToM test based on computerized cartoons (25), possibly due to the smaller sample size in their study (1/3 of our patient sample). In summary, the findings suggest significant impairments of patients with psychosis in both Cognitive ToM and Affective ToM, which once again illustrate the high clinical relevance of different ToM subdomains in psychosis.

With regard to deficits in Cognitive ToM, our results indicate a correlation of medium size between these deficits and general delusions and thus confirmed our hypothesis. However, it should be noted, that in the present study the correlation was based only on one specific PANSS item (P1 general delusions), the interpretation is therefore limited. Unexpectedly, however, there was no association between problems in cognitive ToM and positive symptoms in general, which contradicts the results of some previous studies (21, 25, 26, 51). Interestingly, Blikstedt and colleagues (52) found that first-episode patients with a high level of positive symptoms showed the least severe level of deficits in cognitive ToM, when these patients simultaneously were found to present a lower level of negative symptoms (53). A large number of earlier ToM studies have focused especially on patients' problems in cognitive ToM [e.g. by using the hinting task; (54)] or first/second-order false belief test (55), and some of them found an association between problems in cognitive ToM and positive symptoms (56). Thus, results with regard to associations between cognitive ToM and delusions depend closely on the ToM task and a comparison of the different studies is difficult due to the different task specifics. In addition, meta-analytic evidence of associations between ToM and delusions is also limited, as Ventura and colleagues found an association between cognitive ToM and positive symptoms of small effect size, but did not directly investigate an association between ToM problems and delusions (18).

Concluding, our results suggest that ToM and delusions are associated, but effect sizes seem to be rather small, as several studies did not find an association, possibly due to small sample sizes. This conclusion aligns with the review of Freeman and Garety (16) who criticized that there was little evidence for a specific association between ToM problems due to inconsistent findings, while the results regarding the association between ToM and negative and disorganized symptoms were quite consistent. For this reason, Freeman and Garety even excluded ToM as a possible contributing factor from their current model of the formation and maintenance of (persecutory) delusions (16, 17). In conclusion, Cognitive ToM deficits appear to be overall less associated with delusion or positive symptoms.

In line with our hypotheses, affective ToM was associated with both negative and disorganized symptoms. Studies that assess affective ToM are rare, which limits the possibility to compare our results with other findings. However, our results partially confirm the findings of Shamay-Tsoory (25) and Montag (26), who found an association between deficits in Affective ToM and negative symptoms (assessed using the PANSS and additionally the SANS in the study of Shamay-Tsoory). Contrary to previous findings in correlation studies, our study showed that Affective ToM is associated with disorganized symptoms, which is in line with our hypothesis. Thus, the fact that patients from the symptom cluster with predominantly disorganized symptoms (11, 18) were most severely impaired in their general ToM ability can be partly explained by deficits in Affective ToM.

Moreover, regarding our results, it has to be taken into account that global neurocognitive impairment in psychosis may affect both ToM domains, Cognitive ToM and Affective ToM, as they are related to inferential abilities. It is well known that psychotic patients show neurocognitive impairments, e.g., problems in attention, memory, and IQ (57) besides social-cognitive deficits.

Results of a meta-analysis indicate that both social cognition (e.g., ToM) and neurocognition are associated with functioning (7). Thus, the effects found in the present study may also be partly due to neurocognitive factors rather than psychotic symptoms (58). As we solely assessed verbal IQ with a test that can be viewed as an IQ screening to reduce the burden on the patients, we were not able to control for more global neurocognitive factors. Interestingly, Moritz and colleagues (58) provide several recommendations to address these factors in future studies: they recommend them to “consider mediators that are potentially associated with performance” (e.g., in ToM tasks), second “consider confounder that exists in one group only” (e.g., medication) and third “provide the percentage of participants with impairment.” In summary, this approach could offer insights into the specific associations between social and neurocognitive impairments.

It is remarkable that in the present study, with regard to Hyper-ToM, the patients with psychosis did not differ from non-clinical controls. This finding was evident with and without controlling the effect of verbal IQ. A comparable effect was obtained by Blikstedt (59) and Peyroux (33), who both also found no differences in Hyper-ToM errors between patients with psychosis and non-clinical controls controlling for IQ. Peyroux (33) examined Hyper-ToM errors using the MASC (26), a video-based assessment of reduced Cognitive ToM and Affective ToM and Hyper-ToM approximating real-life social interactions. Interestingly, Montag and colleagues (26) also used the MASC and initially showed that patients with psychotic disorders presented more Hyper-ToM errors compared to non-clinical controls, but the effect did not remain significant after controlling for verbal memory (26). Furthermore, contrary to our hypotheses, we found no association between Hyper-ToM errors and either positive or negative symptoms or disorganized symptoms. However, results from previous studies suggested an association between Hyper-ToM errors and positive symptoms (19, 26, 32, 33) and particularly delusions (19, 26), while disorganization was associated with reduced ToM abilities (32). Although we could not verify these findings, the majority of studies seem to support an association between Hyper-ToM errors and positive symptoms.

Regarding the inconsistent results of Hyper-ToM in psychosis, it has to be taken into account, that we measured Hyper-ToM errors indirectly by analyzing errors, whereas in the MASC, Hyper-ToM is measured directly (26). In the present study, we developed an additional Hyper-ToM scoring (see explanation in the Methods section) and in comparison to the other scores in the study, there was only a small number of opportunities to perform a Hyper-ToM error, which limits our results. Nevertheless, this approach is comparable to other studies, which have also evaluated the Frith-Happé animations test concerning an over-attribution/Hyper-ToM that used a comparable scoring (19, 45). Concluding, our study does not provide evidence for more pronounced Hyper-ToM errors in patients with psychosis. Nevertheless, the research question is only partly solved, as some group differences could be explained by intellectual functioning. Thus, future research should further investigate this question assessing Hyper-ToM errors using direct and reliable assessment methods.

### Clinical Implications

Regarding the clinical implications, our findings indicate that patients with psychotic disorders are impaired in both Cognitive ToM and Affective ToM, which raises the question of how ToM deficits as part of social cognition can be treated. The German guidelines for the treatment of schizophrenia [German Society for Psychiatry and Psychotherapy, Psychosomatics and Neurology; (60)] recommend cognitive remediation training (61) for existing impairments of (social) cognitive abilities. Nevertheless, it is first important to regularly assess social-cognitive deficits such as Cognitive ToM and Affective ToM in patients with psychosis, which is not yet common practice in inpatient and outpatient units in Germany. Second, it is important to start appropriate treatment, e.g., social-cognitive remediation training and/or meta-cognitive training (62).

With regard to the appropriate treatment of ToM deficits, various cognitive remediation training programs on social and neurocognition or cognitive biases in psychotic patients that also aim to reduce ToM deficits have been available for several years: the Social Cognition and Interaction Training (63), the Metacognitive Training (62) and the Integrated Neurocognitive Therapy (64). In general, these training report impressive pre-post effectiveness and are quite successful (61). Several trainings focused specifically on ToM deficits: Emotion and ToM Imitation Training (65); Theory of Mind Intervention (66) and Cognitive-Emotional Rehabilitation (67). In most cases, however, these trainings are primarily offered in psychiatric inpatient treatment, but less often in outpatient units, as mentioned by Moritz and colleagues (68). Furthermore, training of ToM abilities is not part of regular Cognitive Behavioral Therapy for psychosis (69). Thus, to ensure that patients can benefit from the treatment of their ToM difficulties beyond inpatient treatment, it is, therefore, necessary to implement ToM training in outpatient treatment and to combine it with CBTp.

### Strength and Limitations

The present study has several strengths: One strength is the assessment of Cognitive ToM and Affective ToM using the multiple-choice version of the Frith-Happé animations, a validated and reliable measurement (37). As discussed previously, our study is the first study that investigates ToM in psychosis using the more advanced version of the Frith-Happé animations, which evaluates the patients' performance in the test using multiple choice questions and therefore, is indicated more objective by the test authors (37). Furthermore, the social interactive stimuli of the animations have the advantage of being more similar to real-life scenarios, as recommended by Brüne (70). Also, concerning our sample, a size of 64 patients appears to be comparatively large, only seven of 36 studies in a review had a larger sample (17). Furthermore, the heterogeneous characteristics of the patient sample are a strength of the study, as evidenced by both demographic and clinical variables (see Table 1): the age of the patients ranged from 19 to 61 years, the gender was approximately equally distributed (54.7% are female). In addition, as only a small part of the patients reported a first episode of psychosis (*n* = 6), the mean duration of illness was 14 years and the mean number of psychotic episodes was six episodes, we can assume that our sample consists mainly of chronic psychotic patients with severe impairments. Furthermore, one-third of the patients were in current remission and another third of the patients presented acute delusions, which shows that our sample reflects the diversity of actual clinical symptoms in psychosis.

The present study also has some limitations: One limitation is the cross-sectional study design, which does not allow conclusions about the causality of ToM in psychotic disorders. The comparatively small control group (3:1 ratio of patients: controls) represents another limitation. A methodological limitation is the operationalization and measurement of Hyper-ToM errors using the Frith-Happé animations in this study. As discussed previously, the Hyper-ToM score was developed by the authors; thus, a validation of the evaluation has not yet been carried out. Hyper-ToM should therefore be assessed using a more appropriate validated measurement instrument. With regard to the measurement of psychotic psychopathology, the lack of specific assessment of negative symptoms (e.g., BNSS, SANS) is a shortcoming. The interpretation of the correlations between ToM and delusions is limited, as delusions were only measured with one item. A comprehensive measurement instrument regarding delusions [e.g., Psychotic Symptom Rating Scales (PSYRATS), (71)] would allow more reliable statements to be made. In addition, the between-group results might be influenced by problems in neurocognition/IQ. This effect could not be properly controlled by the verbal IQ assessment in the present study, which makes a comprehensive IQ assessment necessary in future studies.

Finally, effects in pre-registered studies are found to be three times smaller than in studies that were not pre-registered (72). Thus, it would be important to replicate our findings provide a more reliable conclusion about the association of ToM dysfunction and symptoms of psychosis.

## Conclusion

Our findings support the established finding of associations between dysfunctions in ToM and negative or disorganized symptoms. Furthermore, the results suggest that deficits in different aspects of ToM may not be directly related to delusions and positive symptoms and are consistent with more recent cognitive models of delusions. However, we found no evidence for more pronounced Hyper-ToM errors in patients with psychosis compared to non-clinical controls and there were no associations between Hyper-ToM errors and psychotic symptoms when controlling for years of education. In sum, our results shed light on the importance of a differentiated consideration of ToM subdomains in the context of psychosis, since the results emphasized the multifaceted relationship of specific ToM dimensions to symptoms in psychosis.

## Data Availability Statement

The raw data supporting the conclusions of this article will be made available by the authors, without undue reservation.

## Ethics Statement

The studies involving human participants were reviewed and approved by Ethics Committee of the Medical Faculty of the Philipps-University Marburg, Germany. The patients/participants provided their written informed consent to participate in this study.

## Author Contributions

LD: conceptualization, methodology, software, formal analysis, investigation, and writing—original draft. NS: recruitment, data collection, and writing—review & editing. FB and IF: writing—review & editing. TK: resources and writing—review & editing. WR: resources, writing—review & editing and supervision. SM: conceptualization, resources, project administration, writing—review & editing, and supervision. All authors contributed to the article and approved the submitted version.

## Conflict of Interest

The authors declare that the research was conducted in the absence of any commercial or financial relationships that could be construed as a potential conflict of interest.
