# Supplementary material for: The Relationship Between Different Aspects of Theory of Mind and Symptom Clusters in Psychotic Disorders: Deconstructing Theory of Mind Into Cognitive, Affective, and Hyper Theory of Mind
Source: Front Psychiatry. 2021 Apr 9;12:607154. doi: 10.3389/fpsyt.2021.607154 (PMC8062806; doi:10.3389/fpsyt.2021.607154)
Supplement: Supplementary file 1 [file Data_Sheet_1.PDF]

Supplements

Additional exploratory analyses

S 1

Comparison of Spearman correlation coefficients between Cognitive ToM, Affective ToM, Hyper-ToM and different PANSS symptom factors in patients with psychosis

			Frith-Happé Cognitive ToM	***Frith-Happé Affective ToM***	***Frith-Happé Hyper-ToM***
	M	SD	r_s (*p*)	r_s (*p*)	r_s (*p*)
PANSS positive symptom factor (Wallwork, et al., 2012)	9.33	3.77	-.196 (.12)	-.188 (.14)	.169 (.22)
PANSS positive symptom factor (Van der Gaag, et al., 2006)	12.16	4.67	-.223 (.08)	-.226 (.08)	.146 (.29)
PANSS negative symptom factor (Wallwork et al., 2012)	12.38	5.04	-.044 (.73)	**-.332 (<.01)**	-.095 (.49)
PANSS negative symptom factor (Van der Gaag, et al., 2006)	15.80	6.13	-.064 (.62)	**-.329 (<.01)**	-.104 (.45)
PANSS negative symptom factor 'expressive deficits' (Liemburg, et al., 2013)	11.11	4.35	-.024 (.85)	**-.392 (.001)**	-.015 (.91)
PANSS negative symptom factor 'social amotivation' (Liemburg, et al., 2013)	6.30	3.20	.012 (.92)	-.156 (.22)	-.175 (.20)
PANSS disorganized symptom factor (Wallwork et al., 2012)	5.48	2.09	-.239 (.06)	**-.286 (.02)**	**.320 (.02)**
PANSS disorganized symptom factor (Van der Gaag, et al., 2006)	8.56	2.66	**-.313 (.01)**	**-.285 (.02)**	**.282 (.04)**

Note: M = Mean; SD = Standard deviation; r_s = Spearman correlation; p = significance; statistical significance is indicated by bold values; PANSS: Positive and Negative Syndrome Scale (Kay et al., 1987)

S 2

Spearman correlation and the partial rank correlation between cognitive ToM, affective ToM, Hyper-ToM and PANSS symptom factors in patients with psychosis

			Frith-Happé Cognitive ToM	*Frith-Happé Affective ToM*	*Frith-Happé Hyper-ToM*
	M	SD	r_s (p)	r_s (p)	r_s (p)
PANSS positive symptom factor (Wallwork, et al., 2012)	9.33	3.77	-.196 (.12)	-.188 (.14)	.169 (.22)
Partial Correlation PANSS positive symptom factor (Wallwork, et al., 2012)	9.33	3.77	-.193 (.14)	-.183 (.16)	.151 (.28)
PANSS negative symptom factor (Wallwork et al., 2012)	12.38	5.04	-.044 (.73)	**-.332 (<.01)**	-.095 (.49)
Partial Correlation PANSS negative symptom factor (Wallwork et al., 2012)	12.38	5.04	-.048 (.72)	**-.331 (<.01)**	-.070 (.62)
PANSS disorganized symptom factor (Wallwork et al., 2012)	5.48	2.09	-.239 (.06)	**-.286 (.02)**	**.320 (.02)**
Partial Correlation PANSS disorganized symptom factor (Wallwork et al., 2012)	5.48	2.09	-.180 (.17)	**-.259 (.04)**	.215 (.12)
Partial Correlation General delusions (PANSS P1)					.187 (.18)
Partial Correlation Persecutory delusions (PANSS P6)					.051 (.72)

Note: M = Mean; SD = Standard deviation; r_s = Spearman correlation; p = significance, two-tailed; statistical significance is indicated by bold values; partial rank correlation is controlled for ‘years of education’.
